# LCZ696, an angiotensin receptor-neprilysin inhibitor, ameliorates epithelial-mesenchymal transition of peritoneal mesothelial cells and M2 macrophage polarization

**DOI:** 10.1080/0886022X.2024.2392849

**Published:** 2024-08-21

**Authors:** Yan Hu, Canxin Zhou, Qin Zhong, Xialin Li, Jinqing Li, Yingfeng Shi, Xiaoyan Ma, Daofang Jiang, Yi Wang, Shougang Zhuang, Na Liu

**Affiliations:** a Department of Nephrology, Shanghai East Hospital, Tongji University School of Medicine, Shanghai, China; b Department of Medicine, Rhode Island Hospital and Alpert Medical School, Brown University, Providence, RI, USA

**Keywords:** LCZ696, epithelial-mesenchymal transition, M2 macrophage polarization, peritoneal fibrosis

## Abstract

**Aims:**

To investigate the effects and mechanisms of LCZ696, an angiotensin receptor-neprilysin inhibitor (ARNI), on epithelial-mesenchymal transition (EMT) of peritoneal mesothelial cells and on macrophage M2 polarization.

**Methods:**

We examined the effects of LCZ696 in a 4.25% high glucose peritoneal dialysis fluid (PDF)-induced peritoneal fibrosis (PF) mouse model, and explored the mechanisms of LCZ696 on human peritoneal mesothelial cells (HPMCs) stimulated by TGF-β1 (5 ng/mL) and on Raw264.7 cells stimulated by IL-4 (10 ng/mL). To further elucidate the mechanism, we treated HPMCs with the conditioned medium of Raw264.7 cells.

**Results:**

LCZ696 effectively improved PF and inhibited the process of EMT in PDF mice. *In vitro*, LCZ696 also significantly alleviated the EMT of TGF-β1 induced HPMCs, although there was no statistically significant difference when compared to the Valsartan treatment group. Moreover, LCZ696 ameliorates the increased expression of Snail and Slug, two nuclear transcription factors that drive the EMT. Mechanistically, TGF-β1 increased the expression of TGFβRI, p-Smad3, p-PDGFRβ and p-EGFR, while treatment with LCZ696 abrogated the activation of TGF-β/Smad3, PDGFRβ and EGFR signaling pathways. Additionally, exposure of Raw264.7 to IL-4 results in increasing expression of Arginase-1, CD163 and p-STAT6. Treatment with LCZ696 inhibited IL-4-elicited M2 macrophage polarization by inactivating the STAT6 signaling pathway. Furthermore, we observed that LCZ696 inhibits EMT by blocking TGF-β1 secretion from M2 macrophages.

**Conclusion:**

Our study demonstrated that LCZ696 improves PF and ameliorates TGF-β1-induced EMT of HPMCs by blocking TGF-β/Smad3, PDGFRβ and EGFR pathways. Meanwhile, LCZ696 also inhibits M2 macrophage polarization by regulating STAT6 pathway.

## 1. Introduction

Peritoneal dialysis (PD) serves as a pivotal form of renal replacement therapy for patients who are suffered from end-stage renal disease (ESRD), which has the characteristics of being economical, better residual renal function preservation and hemodynamically stable [1]. A complete and functional peritoneal membrane ensures the successful of PD [2]. Unfortunately, long-term exposure to bioincompatible PD fluids gradually damage the structural of peritoneal membrane. As a result, up to 50% patients who received PD treatment more than 6 years are prone to peritoneal fibrosis (PF) and even ultrafiltration failure [3]. Therefore, it is crucial to explore the cellular mechanisms facilitating the development of PF.

The pathogenesis of PF is complex and multifactorial. Mounting evidence has indicated that the epithelial-mesenchymal transition (EMT) of the resident peritoneal mesothelial cells plays an important role during fibrogenesis [4]. The process of EMT contributes to the activation of α-SMA and the deposition of collagen, resulting in the abnormal accumulation of extracellular matrix (ECM) in the submesothelial layer [5]. Regarding the underlying molecular mechanisms, ample studies suggest that transforming growth factor-β1 (TGF-β1)/Smads signaling pathway have a central role in the process of PF. The activation of TGFβ receptor can stimulate the phosphorylation of Smads then forming heteromeric complexes. These complexes participate in the process of fibrosis by translocating to the nucleus where they govern gene expression [6, 7]. Moreover, Harald et al. found that a high number of platelet-derived growth factor receptor-β (PDGFRβ) colocalized with α-SMA positive cells were observed in patients under PD therapy [8]. PDGFRβ is a functional player in driving angiogenesis as well as increased collagen gene expression. Overactivation of PDGF contributes to the development of PF [9]. Furthermore, our research group also found that blockade of epidermal growth factor receptor (EGFR) signaling protect the peritoneum from chlorhexidine gluconate and 4.25% peritoneal dialysate fluid (PDF)-induced peritoneal damage [10, 11].

Moreover, emerging evidence has revealed that M2 macrophages are increased in PD effluents as well as peritoneum biopsies of PD patients [12]. Macrophages distributed ubiquitously in many organs and tissues, which differentiate into functionally distinct immunological populations determined by the microenvironment [13]. M2 macrophage polarization (alternatively activated) is a complex dynamic process induced under the stimulation of T helper 2 cells (Th2) cytokines, such as IL-4, IL-10, and IL-13 [14]. The characteristics of M2 macrophages polarization including upregulation of Arginase-1, CD163, CD204, CD206, and, more importantly, produce functional cytokines, such as TGF-β, which could enhance the activation of TGF-β1/Smad3 signaling pathway [15, 16]. Through the aforementioned cytokines, M2 macrophage exerts its pro-fibrotic properties and results in long-term tissue repair and tissue fibrosis [17]. Studies have demonstrated that M2 macrophage polarization promoted the progression of PD-related PF *in vivo* and *in vitro* [18, 19].

Mounting evidence has deduced that the renin-angiotensin-aldosterone system (RAAS) is participated in the process of PF in PD patients and *in vivo* animal models [20, 21]. Clinical studies demonstrated that angiotensin II receptor blockade (ARB), valsartan, can increase peritoneal creatinine clearance [22]. In a chlorhexidine gluconate-induced PF animal model, treatment with ARB and angiotensin-converting enzyme inhibitor (ACEI) significantly decreased the thickness of the submesothelial compact zone [23]. In addition, *in vitro* experiment also found that RAAS components, including angiotensinogen (AGT), renin, angiotensin-converting enzyme (ACE), angiotensin II (ANG II), and the ANG II type 1 receptor (AT1R) were observed in human peritoneal mesothelial cells (HPMCs) [24]. These researches denoted that RAAS involved in the development of PF. Interestingly, LCZ696 (ARNI) is a dual-acting angiotensin receptor neprilysin inhibitor and has been recommended in clinical practice guidelines to treat patients with hypertension or heart failure. The drug combines the valsartan and the neprilysin inhibitor prodrug, sacubitril, in a 1:1 ratio in a sodium supramolecular complex [25]. Sacubitril inhibits neprilysin, the enzyme responsible for the degradation of the natriuretic peptide (NP) system, including atrial NP (ANP), brain NP (BNP) and C-type NP (CNP) [25, 26]. Previous study reported that ANP largely increased the peritoneal fluid removal and small solute clearances by reducing peritoneal fluid absorption [27]. Consistent with this research, Hiroshi et al. demonstrated that ANP ameliorates inflammation-induced PF [28]. Emerging evidence indicated that LCZ696 plays an antifibrotic role in many organs, such as kidney fibrosis [29], liver fibrosis [26], and cardiac fibrosis [30]. However, the effects and mechanisms of LCZ696 in EMT of peritoneal mesothelial cells require elucidation.

In light of the prior research findings, the purpose of the present study was to explore whether LCZ696 would improve PF and ameliorate TGF-β1-induced EMT of peritoneal mesothelial cells and IL-4-induced M2 macrophage polarization.

## 2. Materials and methods

### 2.1. Antibodies and reagents

Antibodies to p-Smad3 (#9520), Smad3 (#9523), E-cadherin (#3195), Snail (#3879), PDGFRβ (#3169), p-PDGFRβ (#3161), STAT6 (#5397), p-STAT6 (#56554), EGFR (#4267) and p-EGFR (#3777) were purchased from Cell Signaling Technology. Antibodies to GAPDH (sc-32233), Collagen I (A2) (sc-28654) and TGFβRI (sc-399) were purchased from Santa Cruz Biotechnology. Antibodies to Slug (ab27568), α-SMA (ab5694), Fibronectin (ab2413) and CTGF (ab6992) were purchased from Abcam (Cambridge, MA). Antibody to Collagen III (A0817) was purchased from ABclonal (Wuhan, China). Antibodies to Arginase-1 (GB11285) and CD163 (GB11340) were purchased from Servicebio (Wuhan, China). Anti-mouse secondary antibody (A0216), and anti-rabbit secondary antibody (A0208) were purchased from Beyotime Institute of Biotechnology (Shanghai, China). TGF-β1 ELISA Kit (RK00057) and IL-4 ELISA Kit (RK00003) were purchased from ABclonal (Wuhan, China). TGF-β1 protein and IL-4 protein were purchased from R&D Systems (Minneapolis, MN, United States). LCZ696 (S7678) was purchased from Selleckchem (Houston, TX, USA). Valsartan (HY-18204) was purchased from MedChemExpress (Shanghai, China).

### 2.2. Animal experiments

Animal experiments were reviewed and approved by the Institutional Animal Care and Use Committee at Tongji University (No. TJBB00624101). C57BL/6J male mice (*n* = 24, 8 weeks old) were purchased from GemPharmatech Co., Ltd. (Nanjing, China). All mice were housed in a 12-h light/dark cycle chamber maintained at a temperature of 23 ± 1 °C, with unrestricted access to food and water. After acclimatizing to the laboratory environment for one week, the mice were randomly allocated into four groups: Sham group, Sham + LCZ696 group, PDF group, PDF + LCZ696 group. The PDF mice were induced by daily intraperitoneal administration of 100 mL/kg of 4.25% high glucose PDF for a period of 28 consecutive days [31], the Sham group and Sham + LCZ696 group were intraperitoneally administered an equal volume of normal saline. To investigate the effect of LCZ696 on PF, LCZ696 (60 mg/(kg·d)) [29] for the Sham + LCZ696 group and PDF + LCZ696 group or vehicle (normal saline) of equal volume for the Sham group and PDF group were administered gastric gavage once a day for 28 days. All mice were euthanized *via* exsanguination under anesthesia induced by inhaling 5% isoflurane mixed with room air at the end of the 28 days duration. Subsequently, the parietal peritoneum was harvested from each mouse for subsequent experimentation.

### 2.3. Cell culture and treatments

The HPMCs and Raw264.7 cells were obtained from the American Type Culture Collection (ATCC, Manassas, VA, USA).

HPMCs were grown in Dulbecco’s modified Eagle’s medium/Nutrient F12 (DMEM/F12) supplemented with 10% fetal bovine serum (FBS) and 1% penicillin/streptomycin in a 5%CO_2_ humidified incubator at 37 °C. To examine the effect and mechanisms of LCZ696 on PF *in vitro*, HPMCs were starved for 24 h with DMEM/F12 containing 0.5% FBS and then pretreated with or without LCZ696 (25 μM) [32] for 1 h and then coincubated with or without TGF-β1 (5 ng/mL) [33] for 36 h before cell harvesting and collection of the culture medium. To compare the effect of Valsartan with LCZ696 on the process of EMT, HPMCs were starved for 24 h with DMEM/F12 containing 0.5% FBS and then pretreated with or without Valsartan (10 μM) [34] for 1 h and then coincubated with or without TGF-β1 (5 ng/mL) for 36 h before cell harvesting.

Raw264.7 cells were grown in RPMI-1640 medium supplemented with 10% FBS and 1% penicillin/streptomycin in a 5%CO_2_ humidified incubator at 37 °C. Raw264.7 cells were starved for 24 h with RPMI-1640 medium containing 0.5% FBS and then pretreated with or without LCZ696 (25 μM) for 1 h and then coincubated with or without IL-4 (10 ng/mL) [35] for 24 h before cell harvesting and collection of the culture medium. The conditioned medium was added to the HPMCs for 36 h before cell harvesting. All of the *in vitro* experiments were repeated for no less than three times.

### 2.4. Immunoblot analysis

Total proteins were extracted from the peritoneum, as well as HPMCs and Raw264.7 cell lysed in radioimmunoprecipitation assay (RIPA) buffer with protease and phosphatase inhibitors on ice. After a centrifugation of 12000 rpm for 15 min at 4 °C, 5× loading buffer of sodium dodecyl-sulfate polyacrylamide gel electrophoresis (SDS-PAGE) were added to the supernatants and denatured at the temperature of 98 °C for 20 min. Equal amounts of proteins were loaded onto and separated by 8%, 10% or 12% SDS-PAGE gels subsequently transferred to Immobilon-P polyvinylidene fluoride (PVDF) membranes (0.22 μm pore size; Millipore, Billerica, MD, USA). The membranes were blocked with 5% skim milk solution for 1 h and underwent an overnight incubation with specific primary antibodies at 4 °C. After extensive washing, the membrane was incubated at ambient temperature with anti-mouse/rabbit secondary antibodies labeled with horseradish peroxidase for 1 h. Finally, the blots were detected using an enhanced chemiluminescence kit (NCM Biotech, Suzhou, China). The expression level was determined by measurement of the corresponding band intensities using ImageJ software (National Institutes of Health, Bethesda, MD, USA).

### 2.5. Masson’s trichrome staining

After fixation in formaldehyde, peritoneum tissues were embedded in paraffin and sectioned into 4-μm slices. These sections were subjected to a 1-h baking process at 65 °C, followed by dewaxing and rehydration. Subsequently, the sections were immersed in reagent A overnight at room temperature according to the manufacturer’s protocol (Servicebio, Wuhan, China). Images were acquired using light microscope (Leica, DM3000). The positive area of Masson’s trichrome staining and thickness of peritoneum were quantitatively measured using Image Pro-Plus software (Media-Cybernetics, Silver Spring, MD, USA).

### 2.6. Immunofluorescence staining

HPMCs and Raw264.7 from different treatment groups were immobilized and incubated with specific primary antibodies against α-SMA, p-STAT6, Arginase-1 and CD163 at the appropriate concentrations at 4 °C overnight. The next day, Texas Red-labeled secondary antibody was added for 1 h at 37 °C. Nuclei were visualized using DAPI staining. Images were acquired using Fluorescence Microscope (Leica, DM3000).

### 2.7. Enzyme-linked immunosorbent assay (ELISA) analysis

The detection of TGF-β1 protein in the culture medium from Raw264.7 cells and IL-4 protein in the culture medium from HPMCs were carried out using ELISA kits supplied by ABclonal (Wuhan, China), following the manufacturer’s protocol.

### 2.8. Statistical analysis

All data are expressed as mean ± standard error of the mean (SEM). The comparisons of individual parameters between two groups were analyzed by Student’s t-test, and differences in multiple groups were compared using one-way analysis of variance. Statistically significant differences between mean values were marked in each graph. Statistical significance was considered at a threshold of *p* < 0.05.

## 3. Results

### 3.1. LCZ696 effectively improved PF and inhibited the process of EMT in PDF-induced mouse model

**Figure 1.**
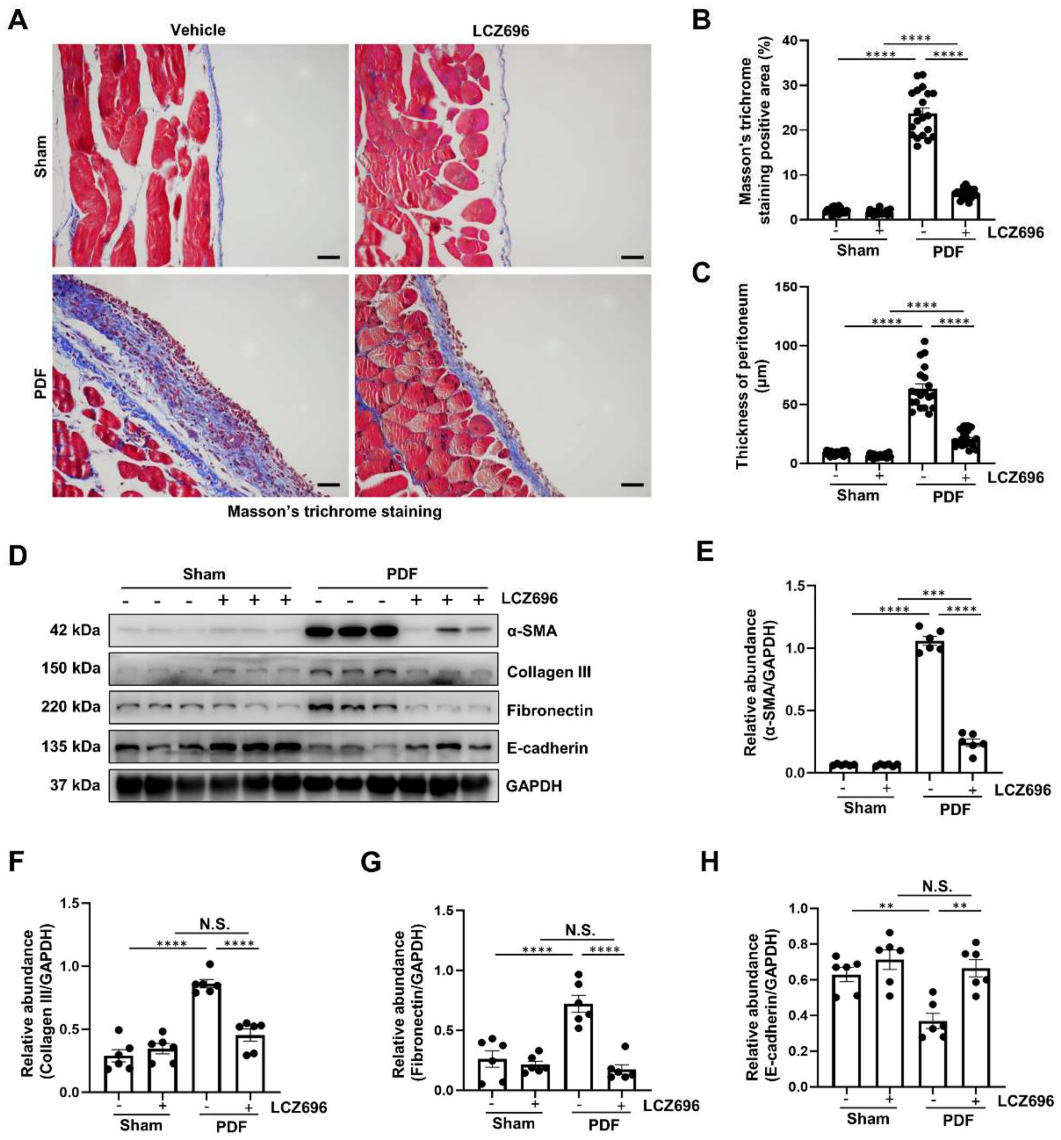
LCZ696 Effectively improved PF and inhibited the process of EMT in PDF-induced mouse model. (A) Photomicrographs illustrating Masson’s trichrome staining of the peritoneum. (B) Scatter plots showing the Masson’s trichrome staining positive area (%). (C) Scatter plots showing the thickness of peritoneum. (D) Western blot was conducted to evaluate the protein level of α-SMA, Collagen III, Fibronectin, E-cadherin and GAPDH in peritoneum. (E–H) Scatter plots showing the densitometry analysis of α-SMA, Collagen III, Fibronectin and E-cadherin normalized by GAPDH. Data are expressed as mean ± SEM. **P < 0.01; ***P < 0.001; ****P < 0.0001. N.S., statistically not significant, with the comparisons labeled. Scale bars in (A) = 50 μm.

Since the protective effect of LCZ696 has been reported in kidney fibrosis [36], cardiac fibrosis [30] and liver fibrosis [26], however, it is unknown whether LCZ696 is protective against peritoneal fibrosis. Here, we established a PDF mouse model to examine the effect of LCZ696 on PF. We found that mice subjected to 28 days of 4.25% PDF injections significantly differed from those in the Sham group, as they exhibited definitive features of PF, including thickening of the submesothelial compact region in the peritoneum, in addition to the accumulation of collagen deposits (Figure 1A). Conversely, the administration of LCZ696 considerably mitigated the increase in peritoneal thickness and inhibited the deposition of collagen (Figure 1A–C). Consistent with the observed histological injury in the peritoneum, the protein expression levels of mesenchymal markers, including α-SMA, Collagen III and Fibronectin were notably elevated in the PDF mice, with the decreased expression of E-cadherin compared to the Sham group (Figure 1D). However, treatment with LCZ696 markedly inhibited the process of EMT (Figure 1D–H). Collectively, these findings demonstrated that LCZ696 effectively improved PF and inhibited the process of EMT *in vivo*.

### 3.2. LCZ696 inhibits TGF-β1-induced EMT of HPMCs

**Figure 2.**
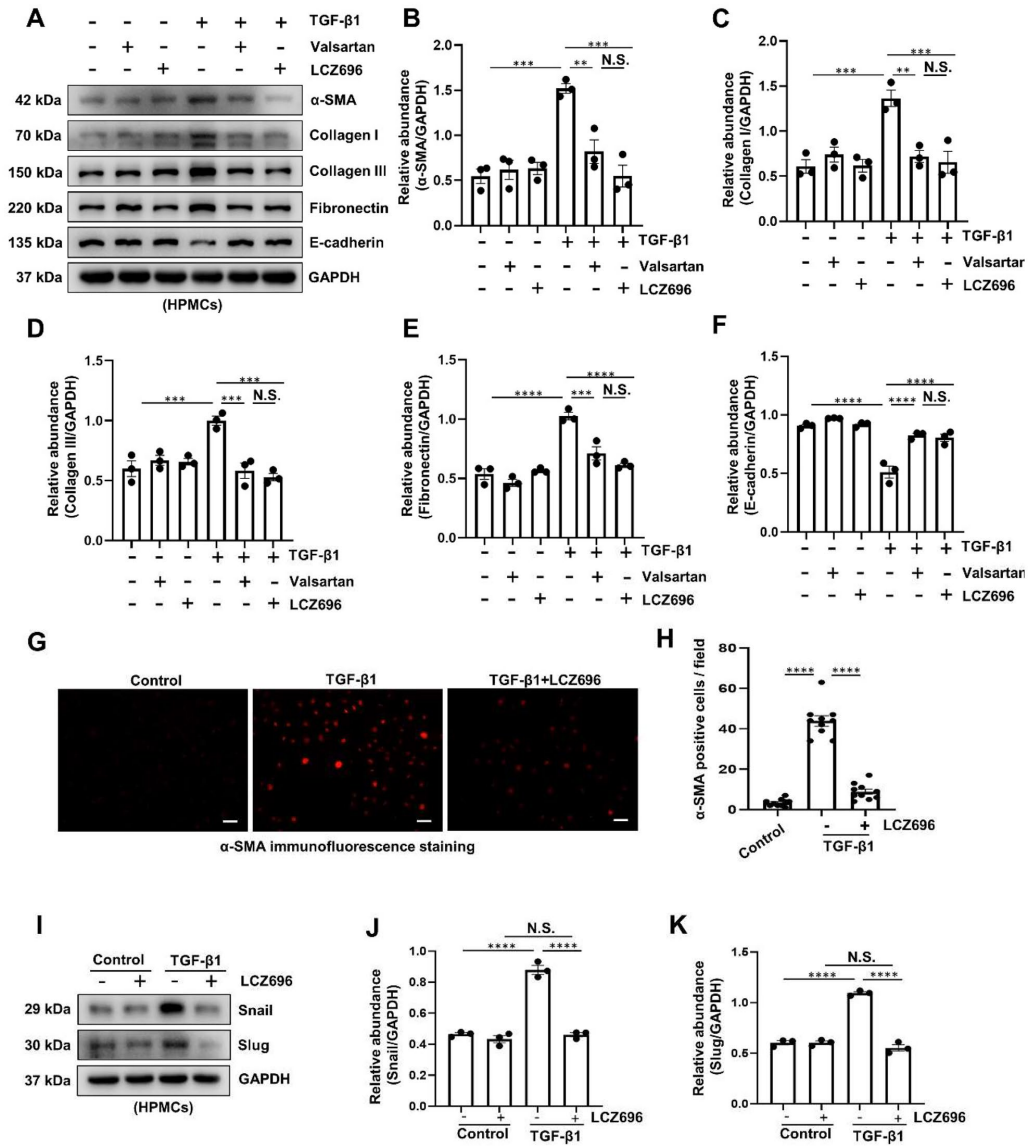
LCZ696 Inhibits TGF-β1-induced EMT of HPMCs. (A) Western blot was conducted to evaluate the protein level of α-SMA, Collagen I, Collagen III, Fibronectin, E-cadherin and GAPDH in HPMCs cell lysates. (B–F) Scatter plots showing the densitometry analysis of α-SMA, Collagen I, Collagen III, Fibronectin and E-cadherin normalized by GAPDH. (G) Photomicrographs illustrating immunofluorescence staining of α-SMA. (H) The count of α-SMA-positive cells. (I) Western blot was conducted to evaluate the protein level of Snail, Slug and GAPDH in HPMCs cell lysates. (J, K) Scatter plots showing the densitometry analysis of Snail and Slug normalized by GAPDH. Data are expressed as mean ± SEM. **P < 0.01; ***P < 0.001; ****P < 0.0001. N.S., statistically not significant, with the comparisons labeled. Scale bars in (G) = 50 μm.

Based on the protective role of LCZ696 on PF *in vivo*, we ­further performed an *in vitro* experiment to illustrate the effects of LCZ696 on TGF-β1-induced EMT of HPMCs. The immunoblot analysis showed that, compared with control group, TGF-β1 significantly increased the expression of α-SMA, Collagen I, Collagen III, Fibronectin, and decreased the expression of E-cadherin. While cotreatment with LCZ696 remarkably downregulation of the mesenchymal markers, and restored the expression of E-cadherin (Figure 2A–F). Notably, while the expression levels of fibrosis-associated proteins in the LCZ696-treated group were diminished compared to those in the Valsartan group, this difference did not reach statistical significance (Figure 2A–F). In addition, immunofluorescence staining of α-SMA further revealed that α-SMA was highly increased induced by TGF-β1, while LCZ696 significantly reduced the elevated expression of α-SMA in HPMCs (Figure 2G,H). Given that Snail and Slug serve as crucial nuclear transcription factors, facilitating the EMT process by repressing the transcription of E-cadherin and other epithelial markers [37], we further investigated the impact of LCZ696 on their expression. Immunoblot analysis showed that exposure of HPMCs to TGF-β1 significantly increased the expression of Snail and Slug, treatment with LCZ696 obviously decreased the protein level of the two nuclear transcription factors (Figure 2I–K). Collectively, these data demonstrated that LCZ696 plays a protective role in TGF-β1-induced HPMCs by ameliorating EMT of HPMCs.

### 3.3. LCZ696 inhibits TGF-β1-induced EMT through the inactivation of the TGFβ/Smad3 signaling pathway

**Figure 3.**
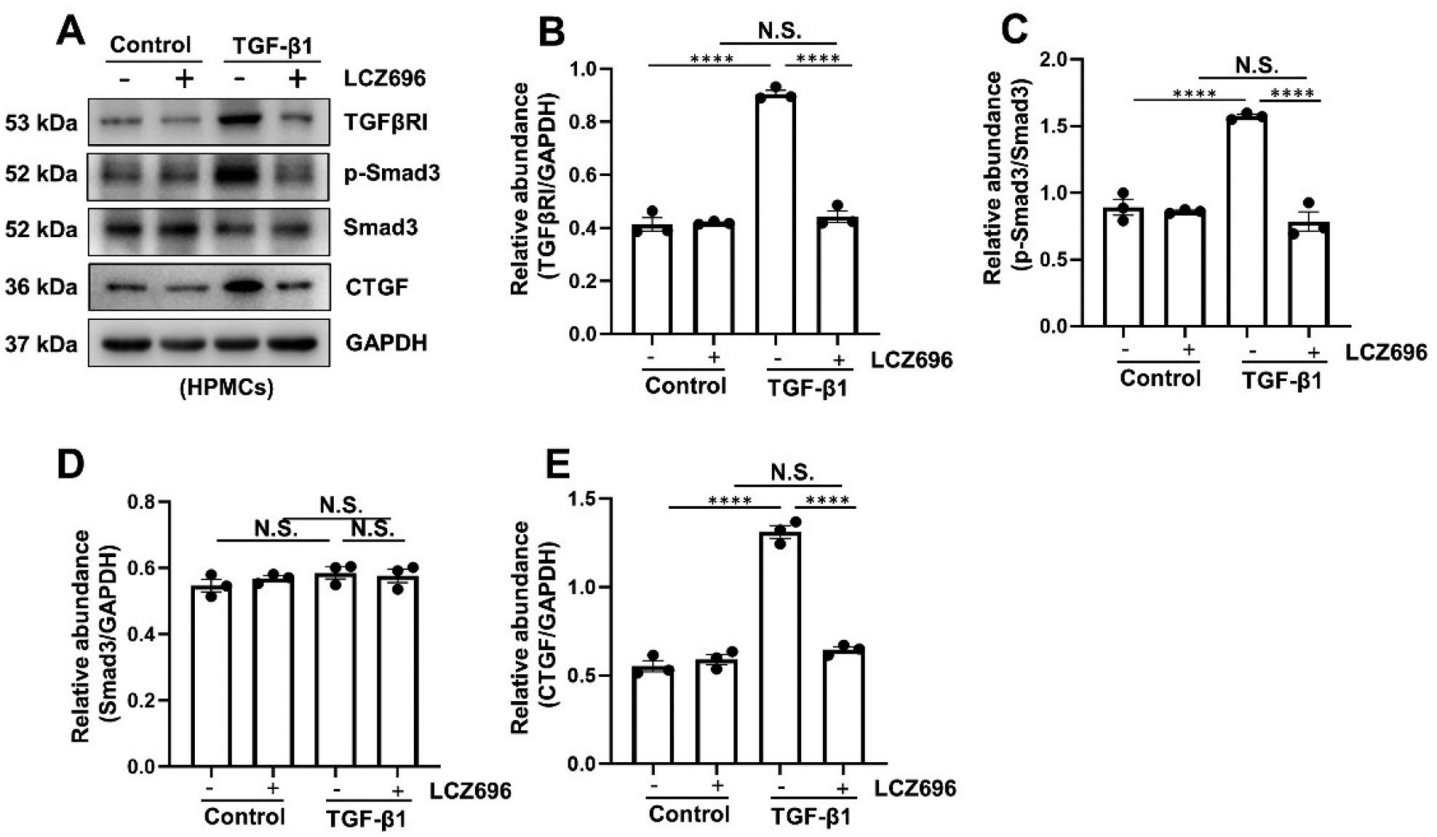
LCZ696 Inhibits TGF-β1-induced EMT through the inactivation of the TGFβ/Smad3 signaling pathway. (A) Western blot was conducted to evaluate the protein level of TGFβRI, p-Smad3, Smad3, CTGF and GAPDH in HPMCs cell lysates. (B, D, E) Scatter plots showing the densitometry analysis of TGFβRI, Smad3 and CTGF normalized by GAPDH. (C) Scatter plot showing the densitometry analysis of p-Smad3 normalized by Smad3. Data are expressed as mean ± SEM. ****P < 0.0001. N.S., statistically not significant, with the comparisons labeled.

Based on the aforementioned results in the study, we further explore the molecular mechanisms of LCZ696 in TGF-β1-induced HPMCs. Regarding the crucial role of TGF-β/Smad3 signaling pathway on the progression of PF [6], we thus evaluated the effect of LCZ696 on this signaling pathway. Western blot results showed that the expression of TGFβRI and p-Smad3 were increased with the TGF-β1 stimulation compared with that in the control group. After LCZ696 administration, the expression level of TGFβRI and p-Smad3 were significantly reduced. However, LCZ696 treatment had no impact on the expression of total Smad3 (Figure 3A–D). We also observed decrease expression of connective tissue growth factor (CTGF), a robust marker of pro-fibrotic factor, under the administration of LCZ696 (Figure 3A,E). Taken together, these data illustrated that LCZ696 inhibits TGF-β1-induced EMT through the inactivation of the TGFβ/Smad3 signaling pathway.

### 3.4. LCZ696 inhibits TGF-β1-induced EMT through the inactivation of the PDGFRβ and EGFR pathways

**Figure 4.**
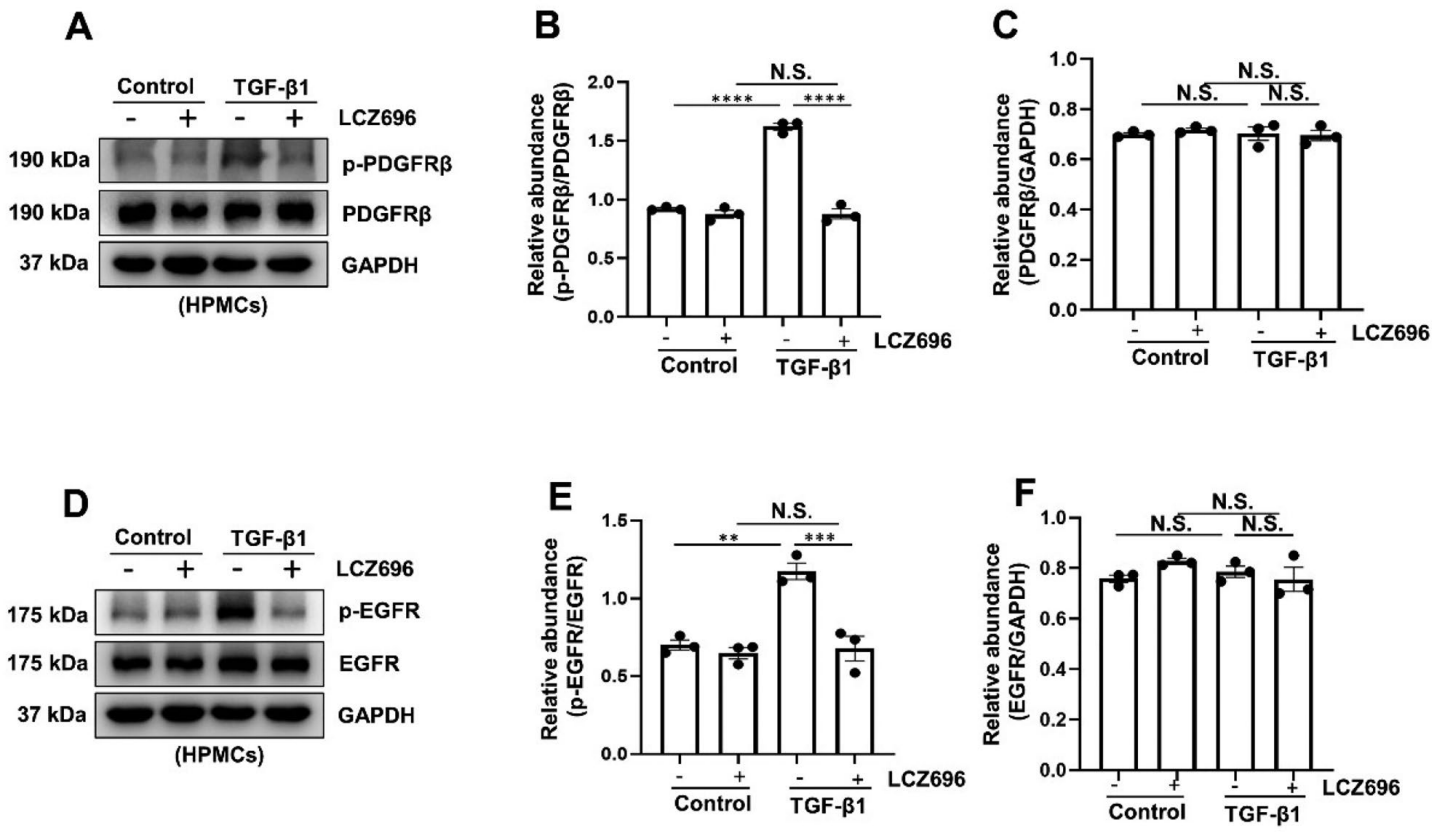
LCZ696 Inhibits TGF-β1-induced EMT through the inactivation of PDGFRβ and EGFR pathway. (A) Western blot was conducted to evaluate the protein level of p-PDGFRβ, PDGFRβ and GAPDH in HPMCs cell lysates. (B) Scatter plot showing the densitometry analysis of p-PDGFRβ normalized by PDGFRβ. (C) Scatter plot showing the densitometry analysis of PDGFRβ normalized by GAPDH. (D) Western blot was conducted to evaluate the protein level of p-EGFR, EGFR and GAPDH in HPMCs cell lysates. (E) Scatter plot showing the densitometry analysis of p-EGFR normalized by EGFR. (F) Scatter plot showing the densitometry analysis of EGFR normalized by GAPDH. Data are expressed as mean ± SEM. **P < 0.01; ***P < 0.001; ****P < 0.0001. N.S., statistically not significant, with the comparisons labeled.

Moreover, it was reported that PDGFRβ and EGFR signaling pathways has been proved to be associated with PF [9, 11]. Thus, we set out to examine the effect of LCZ696 on the activation of PDGFRβ and EGFR pathway. Using immunoblot analysis, we found that the increased of p-PDGFRβ in TGF-β1-stimulated HPMCs were reversed by the treatment of LCZ696, but the expression of total PDGFRβ had no significant difference in each group (Figure 4A–C). In line with this observation, immunoblot analysis demonstrated that LCZ696 also inhibits the phosphorylation of EGFR but had no impact on the expression of total EGFR (Figure 4D–F). Taken together, these data demonstrated that LCZ696 inhibits TGF-β1-induced EMT through the inactivation of PDGFRβ and EGFR pathways in HPMCs.

### 3.5. LCZ696 blocks M2 macrophage polarization by regulating STAT6 signaling pathway

**Figure 5.**
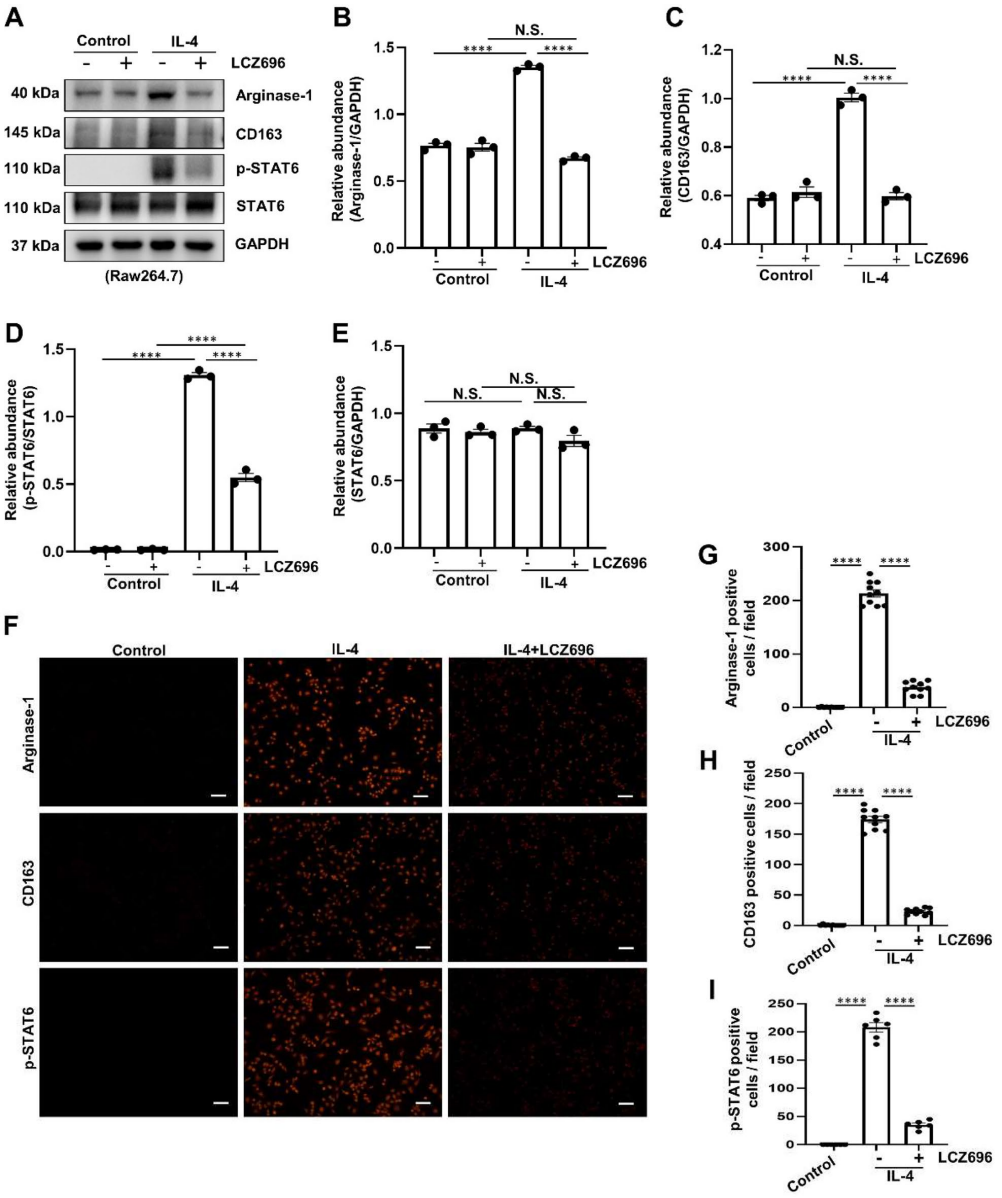
LCZ696 Blocks M2 macrophage polarization by regulating STAT6 signaling pathway. (A) Western blot was conducted to evaluate the protein level of Arginase-1, CD163, p-STAT6, STAT6 and GAPDH in Raw264.7 cell lysates. (B, C, E) Scatter plots showing the densitometry analysis of Arginase-1, CD163 and STAT6 normalized by GAPDH. (D) Scatter plot showing the densitometry analysis of p-STAT6 normalized by STAT6. (F) Photomicrographs illustrating immunofluorescence staining of Arginase-1, CD163, p-STAT6, respectively. (G–I) The count of Arginase-1, CD163, p-STAT6-positive cells. Data are expressed as mean ± SEM. ****P < 0.0001. N.S., statistically not significant, with the comparisons labeled. Scale bars in (F) = 50 μm.

Since a high percentage of M2 macrophage was observed in PD patients [38], we further examined the effect of LCZ696 on IL-4-induced M2 macrophage polarization in Raw264.7, as we all known IL-4 is a robust protein to stimulate macrophage M2 polarization [39]. Both immunoblot and immunofluorescence analysis demonstrated that exposure of Raw264.7 to IL-4 at 10 ng/mL resulted in increased expression of Arginase-1 and CD163, two markers of M2 macrophage. Treatment with LCZ696 was able to suppress the expression of Arginase-1 and CD163 (Figure 5A–C,F–H). Mechanistically, IL-4 induced an increased in the expression of p-STAT6, while LCZ696 inhibited the phosphorylation of STAT6 but did not alter the protein level of total STAT6 (Figure 5), which suggested that LCZ696 may suppress M2 macrophage polarization by regulating STAT6 signaling pathway.

### 3.6. LCZ696 inhibits EMT by blocking TGF-β1 secretion from M2 macrophages

**Figure 6.**
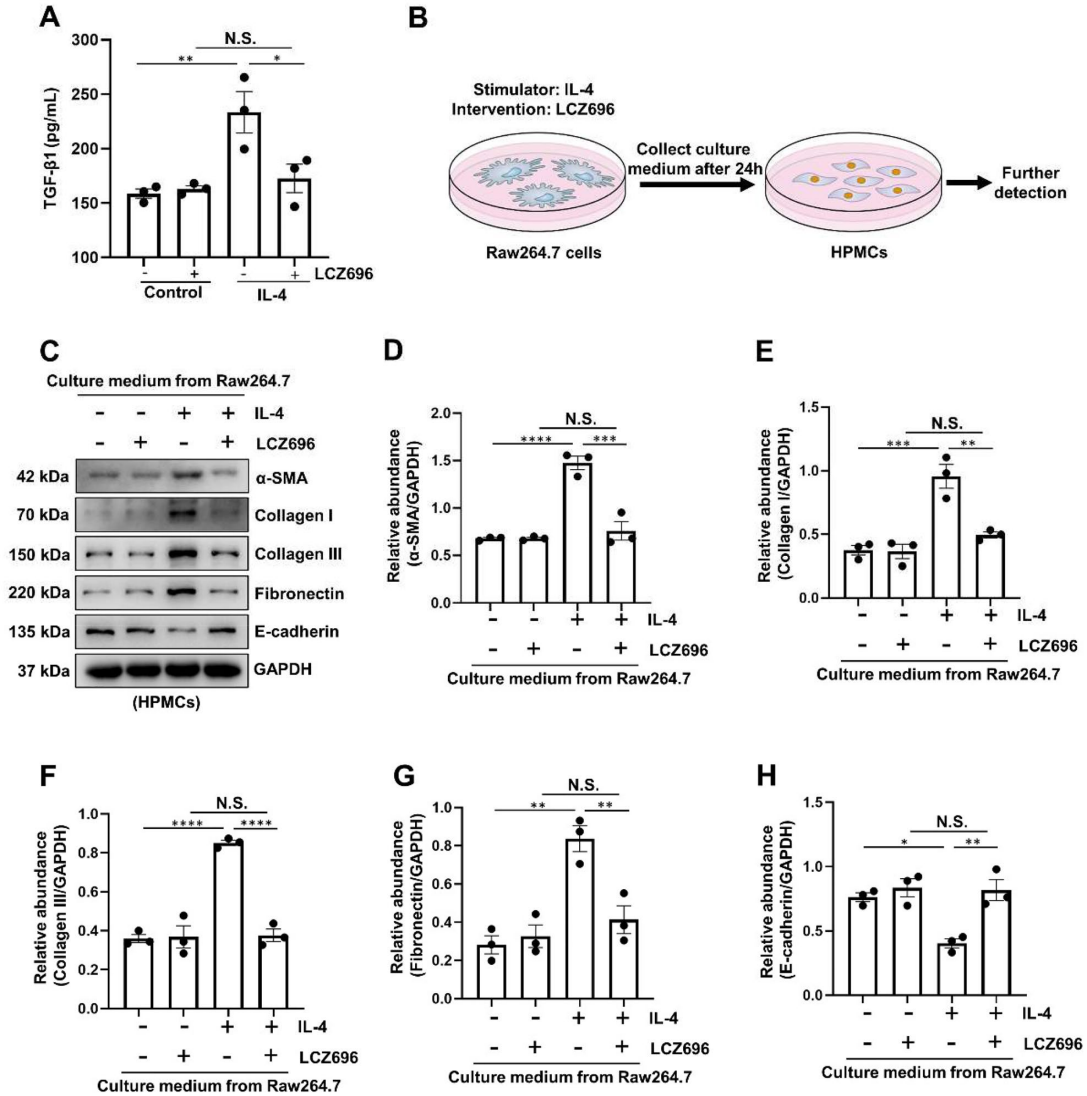
LCZ696 Inhibits EMT by blocking TGF-β1 secretion from M2 macrophages. (A) TGF-β1 level in culture medium of Raw264.7 cells from each group by ELISA kit detection. (B) Experimental scheme for cell treatment: Raw264.7 cells were treated with IL-4 and LCZ696 for 24 hours. Subsequently, the conditioned medium was harvested and used to incubate HPMCs for an additional 36 hours, after which further detections were conducted. (C) Western blot was conducted to evaluate the protein level of α-SMA, Collagen I, Collagen III, Fibronectin, E-cadherin and GAPDH in HPMCs cell lysates. (D–H) Scatter plots showing the densitometry analysis of α-SMA, Collagen I, Collagen III, Fibronectin and E-cadherin normalized by GAPDH. Data are expressed as mean ± SEM. *P < 0.05; **P < 0.01; ***P < 0.001; ****P < 0.0001. N.S., statistically not significant, with the comparisons labeled.

Given that LCZ696 inhibits both peritoneal mesothelial cells’ EMT and M2 macrophage polarization in our study, we further explored the relationship between these two processes. Firstly, we detected the TGF-β1 protein in the culture medium from Raw264.7 cells and IL-4 protein in the culture medium from HPMCs by using ELISA kits. As shown in Figure 6A, a higher level of TGF-β1 was observed in culture medium of Raw264.7 cells stimulated with IL-4, administrated with LCZ696 significantly reduced the secretion of TGF-β1. However, IL-4 was not detected in the culture medium of HPMCs. Therefore, the conditioned medium from Raw264.7 cells was added to the HPMCs for 36 h for further detection (Figure 6B). We found that culture medium from Raw264.7 cells exposed to IL-4 could significantly increase the protein levels of α-SMA, Collagen I, Collagen III, Fibronectin, and decrease the expression of E-cadherin in HPMCs. Culture medium with LCZ696 treatment dramatically reduce the expression of fibrosis-related proteins, and increase the protein level of E-cadherin (Figure 6C–H). Collectively, these data indicated that LCZ696 could inhibit EMT by blocking TGF-β1 secretion from M2 macrophages.

## 4. Discussion

Despite recent progress in cellular mechanisms, there are no effective therapies for peritoneal fibrosis. Recently, LCZ696, a class of new drugs has pleiotropic properties, is reported to performed a robust beneficial role in fibrotic disease, such as renal fibrosis [36], cardiac fibrosis [30] and liver fibrosis [26]. However, the potential effects and mechanisms of LCZ696 on PF are still need more elucidation. In this study, we explored the effects and mechanisms of LCZ696 *in vivo* and *in vitro*. We found that LCZ696 effectively improved PF and inhibited the process of EMT in PDF mice. *In vitro*, LCZ696 also significantly alleviated the EMT of TGF-β1 induced HPMCs. Mechanistically, LCZ696 inhibits TGF-β1-induced EMT through the inactivation of TGF-β/Smad3, PDGFRβ and EGFR signaling pathways. Furthermore, treatment with LCZ696 inhibited IL-4-elicited M2 macrophage polarization by inactivating the STAT6 signaling pathway. LCZ696 also could inhibit EMT by blocking TGF-β1 secretion from M2 macrophages. Our findings provide a theoretical basis for the application of LCZ696 on PF patients.

The pathogenesis of PF is characterized by the accumulation of ECM proteins in peritoneal mesothelial cells. The peritoneal mesothelial cells are the main cellular component of the peritoneum, and the deposition of ECM in peritoneal mesothelial cells are considered to play a pivotal role in the progression of PF [40]. Growing studies indicated that high-glucose peritoneal dialysis fluids could increase the expression of RAAS components, which stimulates the production of TGF-β1 and accumulation of ECM proteins, eventually promotes the development of PF [21, 41]. Treatment with valsartan obviously alleviates ECM accumulation and abrogates PF in high-glucose-induced PF model [34]. On the other hand, intraperitoneal administration of ANP increased peritoneal fluid removal and small solute clearance [27]. Consistently, another research demonstrated that exogenous administration of ANP *via* osmotic pump reduced peritoneal thickening [28]. Therefore, in the present study, we explored the effect of LCZ696, a combination of an ANG II receptor and a neprilysin inhibitor, on the PDF mouse model and TGF-β1-induced HPMCs. We found that administration with LCZ696 effectively improved PF and inhibited the process of EMT *in vivo* and *in vitro*. A similar effect was observed in previous research associate with fibrotic disease. For instance, Junya et al. reported that treatment with sacubitril/valsartan reduced the number of α-SMA-positive myofibroblasts [26]. Zhang et al. revealed that LCZ696 largely attenuated the sloughing of tubular epithelial cells and thickening of the tubular basement membrane in diabetic kidney disease mice [29]. These data suggested that LCZ696 plays a protective role in peritoneal fibrosis.

Basic studies have considered that TGF-β/Smad3 signaling pathway plays a central role in stimulating and aggravating the pathological process of PF [42, 43]. However, the research on whether LCZ696 exert anti-fibrosis role on PF is related to its inactivation of TGF-β/Smad3 signaling pathway are deficiency. In this study, our *in vitro* experiment revealed that LCZ696 inhibits TGF-β1-induced EMT through the inactivation of TGF-β/Smad3 pathway. Consistent with our results, Samah S et al. ­suggested that the effect of LCZ696 to alleviate cyclophosphamide-induced renal fibrosis was obviously superior to that of valsartan, and the protective effect may owe to the ability to downregulate TGF-β/Smad2,3/PAI-1 and NF-κB signaling [44]. Zhang et al. demonstrated that LCZ696 effectively alleviates the cardiac fibrosis through mediating TGF-β1/Smad3 signaling pathway [45]. Mechanistically, a previous study indicated that LCZ696 increases the Smad7, an inhibitory Smad protein, and the upregulation of Smad7 has a negative effect on the activation of Smad3, which can compete with the binding of TGF-β and Smad3 receptor complexes, thus inactivating the process of TGF-β/Smad3 signaling pathway [30]. Nevertheless, the exact mechanism of LCZ696 on PF related to TGF-β/Smad3 signaling pathway needs to be further explored.

In addition, our study also showed that LCZ696 inhibits TGF-β1-induced EMT through the inactivation of PDGFRβ and EGFR signaling pathways in HPMCs. Both PDGFRβ and EGFR are acting an important role in boosting fibroblast and vascular smooth muscle cell proliferation, as well as accumulation of ECM [8, 10]. EGFR, a member of the ErbB family of receptor tyrosine kinases, plays a pivotal role in regulating cell proliferation and migration [46]. Dysregulated EGFR signaling can lead to EMT and tumorigenesis [47]. Our previous study demonstrated that blockade of EGFR signaling pathway protect the peritoneum from two animal models of PF [10, 11]. Treatment with EGFR inhibitor Gefitinib blocks TGF-β/Smad3 signaling pathway in a rat model of PF induced by chlorhexidine gluconate (CG). Delayed administration of Gefitinib also inhibits activation of the TGF-β/Smad3 signaling pathways in the peritoneum after CG injury. In addition, treatment with gefitinib blocks TGF-β1 induced phosphorylation of EGFR and Smad3 in HPMCs [10]. Our previous results imply that the EGFR pathway is situated upstream of the TGF-β/Smad3 pathway. Future studies aimed at dissecting the precise relationship, including possible feedback loops and crosstalk, would be instrumental. Moreover, the activation of PDGFR pathway stimulated the process of partial EMT in a murine model of PF and activated the HPMCs proliferation [48, 49]. In the contrary, silence the signal of PDGFR improved PF [9]. However, studies have shown that inhibition of neprilysin in pulmonary artery smooth muscle cells could excessive activation of the cytoplasmic Src kinase and ­phosphate and tensin homologue (PTEN) phosphorylation, which eventually leads to PDGFR activation [50, 51]. The controversy between this research and our present study may attribute to the different cell types were using in the experiment. Thus, further *in vivo* research with animal models or clinical trials will be beneficial to elucidate the effect of LCZ696 in PDGFR.

According to reported literatures, macrophage M2 polarization involved in the development of PF, and the reduction of M2 macrophages could alleviate the thickening of sub-mesothelial compact zone in PF animal model [52]. Meanwhile, CD68-positive macrophage cells were observed in the peritoneum of PF murine model [31]. M2 macrophage polarization participated in the process of PF may attribute to its role in fibrotic remodeling and tissue repair function [53]. In the present study, we revealed that treatment with LCZ696 was able to suppress the expression of Arginase-1 and CD163, two markers of M2 macrophage polarization. Mechanistically, the interaction of IL-4 with macrophage surface receptors prompts the dimerization of the IL-4Ra and γ chains. This activation triggers cross-phosphorylation at Janus kinases JAK1 and JAK3, which are associated with the receptor. Subsequently, the phosphorylated IL-4Ra receptor attracts and recruits STAT6 *via* its SH2 structural domain. JAK1 then phosphorylates the recruited STAT6. The p-STAT6 is released into the cytoplasm, where it dimerizes and subsequently translocate to the nucleus. Within the nucleus, it initiates the transcription of M2 macrophage-related genes, such as Arginase-1 [54]. As expected, we also found that LCZ696 inhibited the phosphorylation of STAT6. These results were consistent with our previous research [19]. Accordingly, these data indicated that LCZ696 may suppress M2 macrophage polarization by regulating STAT6 signaling pathway. On the other hand, numerous studies revealed that M2 macrophage exerts its fibrotic role through TGF-β/Smad3 signaling pathway [52, 55]. In the current study, a higher level of TGF-β1 was observed in culture medium of Raw264.7 cells stimulated with IL-4, administrated with LCZ696 significantly reduced the secretion of TGF-β1. In addition, we found that culture medium from Raw264.7 cells exposed to IL-4 could significantly increase the protein levels of α-SMA, Collagen I, Collagen III, Fibronectin, and decrease the expression of E-cadherin in HPMCs. Culture medium with LCZ696 treatment dramatically reduce the expression of fibrosis-related proteins, and increase the protein level of E-cadherin. Collectively, these data indicated that LCZ696 could inhibit EMT by blocking TGF-β1 secretion from M2 macrophages.

In conclusion, of particular interest is the fact that the current study presents *in vivo* and *in vitro* evidence of LCZ696’s ability to inhibit peritoneal fibrosis. The cellular mechanisms of LCZ696 on TGF-β1-induced EMT of peritoneal mesothelial cells may through mediating TGF-β/Smad3, PDGFRβ and EGFR signaling pathways. In addition, LCZ696 ameliorates IL-4-induced M2 macrophage polarization *via* regulating STAT6 signaling pathway. LCZ696 also could inhibit EMT by blocking TGF-β1 secretion from M2 macrophages. Although LCZ696 has been identified as a first-in-class inhibitor for the treatment of hypertension or heart failure, our results suggested that this drug may also effective in treatment peritoneal fibrosis. Nevertheless, further research and clinical trials are needed to confirm the antifibrotic effects of LCZ696 on peritoneal fibrosis.
